# Supplementary material for: CARE as a wearable derived feature linking circadian amplitude to human cognitive functions
Source: NPJ Digit Med. 2023 Jul 11;6:123. doi: 10.1038/s41746-023-00865-0 (PMC10336141; doi:10.1038/s41746-023-00865-0)
Supplement: Supplementary file 1 — Reporting Summary [file 41746_2023_865_MOESM1_ESM.pdf]

nature portfolio

Corresponding author(s): Xiaolei Wang, Xinyue Li, Fan Jiang

Last updated by author(s): Feb 6, 2023

Reporting Summary

Nature Portfolio wishes to improve the reproducibility of the work that we publish. This form provides structure for consistency and transparency in reporting. For further information on Nature Portfolio policies, see our Editorial Policies and the Editorial Policy Checklist.

Statistics

For all statistical analyses, confirm that the following items are present in the figure legend, table legend, main text, or Methods section.

n/a	Confirmed	
☐	☒	The exact sample size (*n*) for each experimental group/condition, given as a discrete number and unit of measurement
☐	☒	A statement on whether measurements were taken from distinct samples or whether the same sample was measured repeatedly
☐	☒	The statistical test(s) used AND whether they are one- or two-sided<br>*Only common tests should be described solely by name; describe more complex techniques in the Methods section.*
☐	☒	A description of all covariates tested
☐	☒	A description of any assumptions or corrections, such as tests of normality and adjustment for multiple comparisons
☐	☒	A full description of the statistical parameters including central tendency (e.g. means) or other basic estimates (e.g. regression coefficient) AND variation (e.g. standard deviation) or associated estimates of uncertainty (e.g. confidence intervals)
☐	☒	For null hypothesis testing, the test statistic (e.g. *F*, *t*, *r*) with confidence intervals, effect sizes, degrees of freedom and *P* value noted<br>*Give P values as exact values whenever suitable.*
☒	☐	For Bayesian analysis, information on the choice of priors and Markov chain Monte Carlo settings
☒	☐	For hierarchical and complex designs, identification of the appropriate level for tests and full reporting of outcomes
☐	☒	Estimates of effect sizes (e.g. Cohen's *d*, Pearson's *r*), indicating how they were calculated

Our web collection on statistics for biologists contains articles on many of the points above.

Software and code

Policy information about availability of computer code

Data collection	The UK Biobank data was downloaded using ukbfetch software and the conversion was done by ukbconv software.
Data analysis	Accelerometer data analysis: R (v 4.1.2);<br>Melatonin data analysis: Matlab 2020a;<br>CARE calculation: R package Rssa (v 1.0.4);<br>Genome-wide analysis: PLINK (v1.9 & v2.0);<br>Estimation of heritability of CARE: LDSC (v1.0.1);<br>Two-sample Mendelian randomisation analysis: MendelianRandomisation (v0.5.6);<br>Single-tissue and Cross-tissue Transcriptome-wide Association Analysis: UTMOST;<br>Codes for calculating CARE and relative amplitude were publicly available at https://github.com/Tanyayaya/Circadian-activity-rhythm-energy.

For manuscripts utilizing custom algorithms or software that are central to the research but not yet described in published literature, software must be made available to editors and reviewers. We strongly encourage code deposition in a community repository (e.g. GitHub). See the Nature Portfolio guidelines for submitting code & software for further information.

Data

Policy information about availability of data

All manuscripts must include a data availability statement. This statement should provide the following information, where applicable:

- Accession codes, unique identifiers, or web links for publicly available datasets
- A description of any restrictions on data availability
- For clinical datasets or third party data, please ensure that the statement adheres to our policy

The melatonin and adolescent datasets used in the current study are not publicly available but are available from the corresponding author (Fan Jiang) on reasonable request. The UK Biobank data that support the findings of this study are available from the UK Biobank project but restrictions apply to the availability of these data, which were used under license for the current study (application number: 57947), and so are not publicly available.

Human research participants

Policy information about studies involving human research participants and Sex and Gender in Research.

Reporting on sex and gender	Our analysis includes biological sex as a factor and there was no gender-sex difference in the participants recruited in this study.
Population characteristics	The melatonin dataset: 33 individuals (17 male and 16 female aged 23-61 years).<br>The adolescent dataset: 1703 individuals aged 10 - 19.<br>The adult dataset: 92202 participants aged 56.16 (7.82) from the UK Biobank cohort.
Recruitment	The melatonin dataset recruited 33 healthy adults (17 male and 16 female) aged 23-61 years without reported sleep disorders or shift work.<br>The adolescent dataset used a subsample from the Shanghai Children's Health, Education and Lifestyle Evaluation-Adolescent (SCHEDULE-A).<br>The adult dataset used data from the UK Biobank cohort.
Ethics oversight	The melatonin dataset was approved by the Shanghai Children's Medical Center’s Human Ethics Committee (SCMCIRB-K2021070-2) and Shanghai Jiao Tong University School of Medicine (SJTUPN-202301).<br>The SCHEDULE-A dataset was approved by the Human Ethics Committee of the Shanghai Children's Medical Center according to the Declaration of Helsinki (SCMCIRB-K2018103).<br>The UK Biobank has received ethical approval from the North-West Research Ethics Committee (11/NW/03820) .

Note that full information on the approval of the study protocol must also be provided in the manuscript.

Field-specific reporting

Please select the one below that is the best fit for your research. If you are not sure, read the appropriate sections before making your selection.

☒ Life sciences ☐ Behavioural & social sciences ☐ Ecological, evolutionary & environmental sciences

For a reference copy of the document with all sections, see nature.com/documents/nr-reporting-summary-flat.pdf

Life sciences study design

All studies must disclose on these points even when the disclosure is negative.

Sample size	The melatonin dataset: n=33;<br>The adolescent dataset: n=1703;<br>The adult dataset: n=92202;<br>GWAS study of circadian energy from the UK Biobank: n=85361.<br>Sample sizes were determined based on maximum available data that met the inclusion criteria. No specific statistical methods or sample size estimations were used to define sample size.
Data exclusions	For Chinese SCHEDULE-A, participants with accelerometer data for < 3 days were excluded.<br>For the UK Biobank study, participants were excluded if their accelerometer data were identified by the UK Biobank as poorly calibrated (n = 11) or unreliable (unexpectedly small or large size, n = 4,692) or insufficient wear time (n = 4,465).<br>For GWAS study in the UK Biobank study, we excluded variants based on quality control metrics, such as MAF, imputation quality, and excluded samples from non-European ancestry described in the Methods section of the manuscript.
Replication	We evaluated the correlations between our wearable-derived measure and cognitive functions in independent datasets in the adolescent (Chinese SCHEDULE-A) and adult populations (the UK Biobank).

Randomization	Participants of the melatonin dataset were recruited by convenience sampling. Participants of the adolescent dataset were selected using a multi-stage random sampling approach.
Blinding	All the three datasets we used were observational studies, so blinding of the experimental design does not apply.

Reporting for specific materials, systems and methods

We require information from authors about some types of materials, experimental systems and methods used in many studies. Here, indicate whether each material, system or method listed is relevant to your study. If you are not sure if a list item applies to your research, read the appropriate section before selecting a response.

Materials & experimental systems

n/a	Involved in the study
☒	☐ Antibodies
☒	☐ Eukaryotic cell lines
☒	☐ Palaeontology and archaeology
☒	☐ Animals and other organisms
☒	☐ Clinical data
☒	☐ Dual use research of concern

Methods

n/a	Involved in the study
☒	☐ ChIP-seq
☒	☐ Flow cytometry
☒	☐ MRI-based neuroimaging
