# Supplementary material for: CARE as a wearable derived feature linking circadian amplitude to human cognitive functions
Source: NPJ Digit Med. 2023 Jul 11;6:123. doi: 10.1038/s41746-023-00865-0 (PMC10336141; doi:10.1038/s41746-023-00865-0)
Supplement: Supplementary file 2 — Supplementary materials [file 41746_2023_865_MOESM2_ESM.pdf]

Supplementary Figures

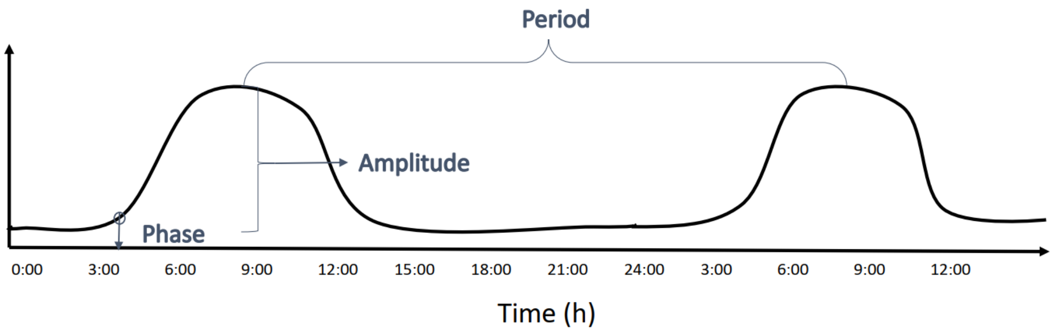

Supplementary Figure 1. Illustration of period, phase and amplitude of circadian rhythms.

Period is the length of a cycle, specifically, it is the time interval between two reference points within a recurring wave (for instance, between hormonal peaks). Phase is defined as the timing of a reference point in the cycle relative to a fixed event. In relation to the melatonin secretion cycle, for example, dim light melatonin onset (DLMO) is the gold standard for the phase of melatonin rhythms. Amplitude is defined as difference between crest and trough values of the cycle. In relation to the hormonal cycle, it would be the difference between the trough and peak hormone levels within a time period (i.e., 24 hours).

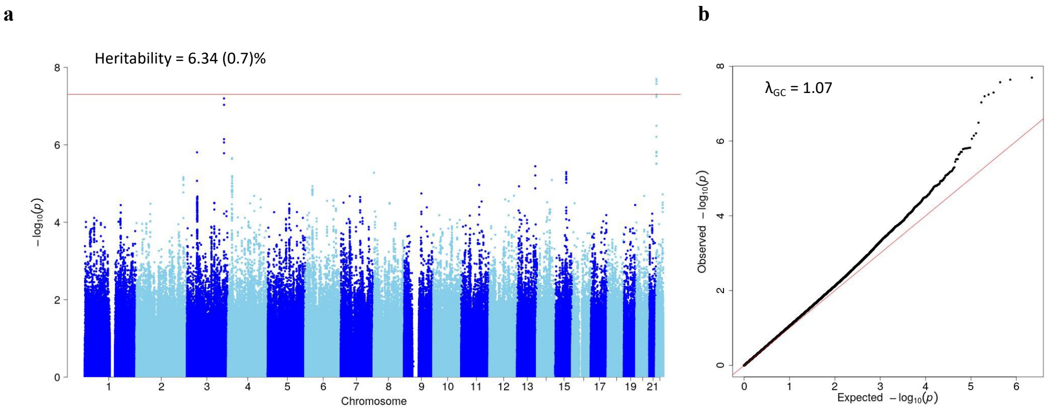

Supplementary Figure 2. Manhattan and QQ plots for relative amplitude associated-SNPs in the adult dataset (UK Biobank) (n = 85,361).

(**a**) The Manhattan plot shows association test (-$\log_{10}$ *P*-value on the y-axis against physical autosomal location on the x-axis). The red line represents genome-wide significance ($P < 5\times10^{-8}$). Heritability estimate was calculated using LDSC tool. (**b**) The QQ plot identifies a slight inflation (λ_{GC} = 1.07) in the test statistic.

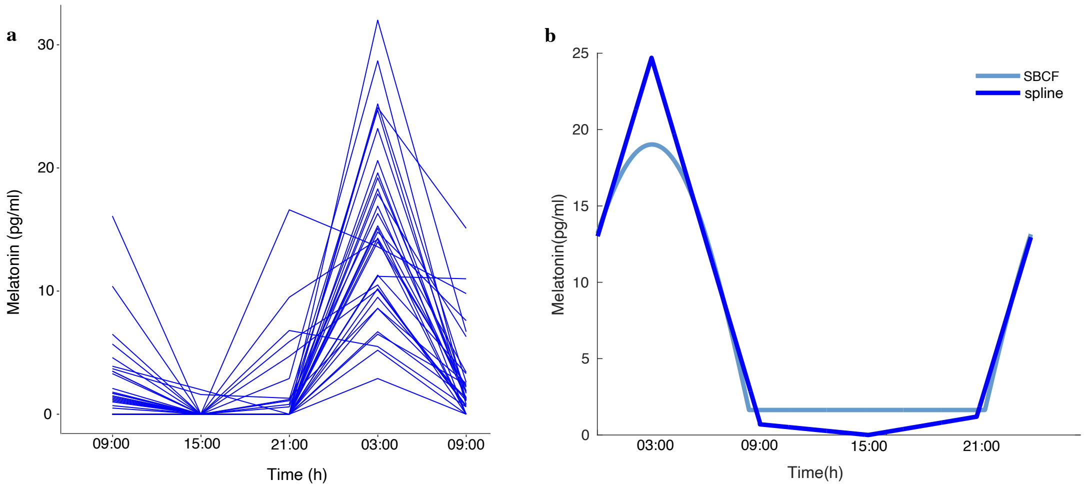

Supplementary Figure 3. The melatonin profiles in the melatonin dataset.

(**a**) Subject-level melatonin profiles (observed values) in the melatonin dataset. Saliva melatonin were collected for five times (i.e., 9 am, 15 pm, 21 pm, 3 am, and 9 am of the next day; time window ± 1 hour) during 24 hours for each participant. (**b**) Illustration of the daily melatonin profile of an individual. Five melatonin sample points were first linear interpolated and then fitted with the skewed baseline cosine function (SBCF) model to get the melatonin secretion curve. Melatonin amplitude was calculated by subtraction of the maximum and minimum values of the melatonin profiles.

Supplementary Tables

Supplementary Table 1. Associations between CARE and melatonin amplitude in the melatonin dataset.

Covariates	Coefficient	SE	*P* value	Variance explained
CARE	58.38	20.24	0.007	21.16%
age	-0.15	0.12	0.21	4.3%
sex	-0.22	2.15	0.92	0.03%

CARE = circadian activity rhythm energy, SE = standard error. Linear regression analysis was performed to examine the association between CARE and melatonin amplitude, adjusted for age and sex in the melatonin dataset. The significant level was set as $P < 0.05$.

Supplementary Table 2. Description of the analyzed variables in the adolescent dataset (Chinese SCHEDULE-A).

Variables	Mean ± SD / n(%)
Device-derived features of circadian amplitude	
CARE	0.10 ± 0.04
Relative amplitude	0.91 ± 0.05
Cognitive scores	
BRI	48.86 ± 9.22
MI	51.60 ± 9.80
GEC	50.63 ± 9.71
Other confounders	
Parental education level	
Lower than high school	426 (25.0)
High school	482 (28.3)
College or higher	795 (46.7)
Income level	
<50,000 CNY	432 (25.4)
50,000 ~ 150,000 CNY	765 (44.9)
⩾150,000 CNY	506 (29.7)
Main caregiver	
parents	1562 (91.7)
grandparents or others	141 (8.3)

BRI = Behavioral Regulation Index, CARE = circadian activity rhythm energy, GEC = Global Executive Composite, MI = Metacognition Index, SD = standard deviation.

Supplementary Table 3. Description of the analyzed variables in the adult dataset (UK Biobank).

Variables	Mean ± SD / n(%)
Device-derived features of circadian amplitude	
CARE	0.13 ± 0.04
Relative amplitude	0.87 ± 0.06
Cognitive scores	
Processing/reaction speed	545.50 ± 105.36
Fluid intelligence scores	7.58 ± 2.07
Reasoning ability	4.89 ± 0.72
Prospective memory results (number of attempts)	
1	29455 (86.2)
2	4735 (13.9)
Other confounders	
Townsend scores	-1.73 ± 2.82
BMI (kg/m^2)	26.71 ± 4.53
Average daily activity intensity	27.99 ± 8.24
Ethnicity	
White	89045 (96.6)
Non-white	3157 (3.4)
Qualification	
Below college	39587 (42.9)
College or higher	52070 (56.5)
Smoking status	
Never	52532 (57.0)
Previous	33090 (35.9)
Current	6334 (6.9)
Frequency of alcohol intake	
Never	5212 (5.7)
Regularly ('1-2 times a week' /'3-4 times a week')	47059 (51.0)
Occasionally ('1-3 times a month' / 'special occasions only')	18796 (20.4)
Daily ('daily' / 'almost daily')	21058 (22.8)
Season at the time when accelerometer monitoring started	
Spring	20032 (21.7)
Summer	19663 (21.3)
Autumn	24477 (26.5)
Winter	28030 (30.4)

CARE = circadian activity rhythm energy, SD = standard deviation.

Supplementary Table 4. The intra-subject and inter-subject variability of CARE values in the adolescent and adults dataset.

	Df	Sum Sq	Mean Sq	F value	*P* value (>F)
Adolescent					
Subject	999	2.00	0.002	3.71	<0.0001
Repetition	1	0.001	0.001	1.76	0.18
Residuals	999	0.54	0.001		
Adult					
Subject	999	2.93	0.003	2.34	<0.0001
Repetition	1	0.002	0.002	1.34	0.25
Residuals	999	1.25	0.001		

CARE = circadian activity rhythm energy, Df = degrees of freedom, Mean Sq = mean square, Sum Sq = sum of squares. An analysis of variance (ANOVA) was performed in a subset of 1,000 individuals each from the adolescent and adult dataset who had at least six days of accelerometer data, and the significance level for the ANOVA was $P < 0.05$.

Supplementary Table 5. The between-group variability of CARE values in the adult dataset.

	Df	Sum Sq	Mean Sq	F value	*P* value (>F)
Group	3	0.02	0.006	3.39	0.02
Residuals	38752	66.39	0.002		

CARE = circadian activity rhythm energy, Df = degrees of freedom, Mean Sq = mean square, Mean Sq = mean square. An analysis of variance (ANOVA) was performed in individuals with psychiatric disorders, such as bipolar affective disorder (n = 147), schizophrenia(n = 42), depression (n = 2,252), and in a control group (n = 36,315) from the adult dataset, and the significance level for the ANOVA was $P < 0.05$.

Supplementary Table 6. Associations between relative amplitude and cognitive functions in the adolescent study (SCHEDULE-A).

Cognitive Scores	n	Coefficient	SE	*P* value
BRI	1,703	-7.13	7.65	0.35
MI	1,703	-14.82	7.26	0.04
GEC	1,703	-11.65	5.61	0.04

BRI = Behavioral Regulation Index, GEC = Global Executive Composite, MI = Metacognition Index, SE = standard error. Median regression models were used with adjusting age, sex, parental education level, family income, and primary caregiver. The significance level was set at $P < 0.017$.

Supplementary Table 7. Associations between relative amplitude and cognitive functions in the adult dataset (UK Biobank).

Cognitive Scores	n	Coefficient/OR	SE	*P* value
**Processing/reaction speed**	91,830	- 41.88	5.22	<0.0001
**Reasoning ability**	34,656	1.30	0.23	0.08
**Short-term memory**	77,439	0.95	0.11	0.64
**Prospective memory**	34,173	1.10	0.37	0.71

OR = odds ratio, SE = standard error. Models were adjusted for age, sex, ethnicity, Townsend score, and the season when the participant started wearing the accelerometer. Linear regression for processing/reaction speed, ordinal logistic regression for reasoning ability and short-term memory scores, and logistic regression for prospective memory were employed. The significance level was set at $P < 0.013$.

Supplementary Table 8. Lead variants associated with CARE in the adult dataset (UK Biobank).

SNP	Chr:position	Nearest gene(s)	Alleles (E/A)	BETA	SE	*P* value
3_49673081_ CCGGG_C	3:49673081	BSN, APEH	CCGGG/C	-0.001	0.0002	1.67×10^{-9}

CARE = circadian activity rhythm energy, Chr = chromosome, E/A = effect/non-effect alleles, position = base pair coordinate hg38, SE = standard error, SNP = single nucleotide polymorphism. Genetic association analysis was performed in related subjects of European ancestry using linear regression models adjusted for age, sex, 20 principal components of ancestry, genotyping array, and genetic correlation matrix. Only lead variants in each locus are shown above. Genes indicate all genes within the locus of interest.

Supplementary Table 9. Summary GWAS statistics for CARE in the adult dataset (UK Biobank).

Index	CHR	SNP	POS	A1	A2	BETA	SE	*P* value
1	3	rs13086611	49347984	T	A	-0.00108932	0.000198838	4.30×10^{-8}
2	3	3:49386313_AAAAAGAAAAAG_A*	49348880	A	AAAAAGAAAAAG	-0.00111922	0.000198991	1.87×10^{-8}
3	3	rs17080528	49352409	T	C	-0.00110056	0.0001986	3.01×10^{-8}
4	3	rs13090388	49353649	T	C	-0.00109841	0.00019855	3.17×10^{-8}
5	3	rs11711536	49353807	T	C	-0.00111388	0.000198775	2.10×10^{-8}
6	3	rs1050450	49357401	A	G	-0.00110774	0.000198313	2.33×10^{-8}
7	3	rs1800668	49358324	A	G	-0.00112189	0.000198216	1.52×10^{-8}

8	3	rs3811699	49358927	C	T	-0.00111665	0.000198181	1.76×10^{-8}
9	3	rs1987628	49361826	A	G	-0.00112189	0.000198216	1.52×10^{-8}
10	3	rs67485053	49367731	G	A	-0.00111777	0.000198203	1.71×10^{-8}
11	3	rs148734725	49369275	A	G	-0.00111069	0.000198237	2.12×10^{-8}
12	3	rs34588335	49370227	C	G	-0.00111616	0.000198178	1.79×10^{-8}
13	3	rs13087851	49371541	G	A	-0.00111546	0.000198202	1.83×10^{-8}
14	3	rs6809216	49375126	A	G	-0.00111477	0.000198172	1.86×10^{-8}
15	3	rs7623659	49377358	T	C	-0.00112135	0.000198289	1.56×10^{-8}
16	3	rs7648841	49379392	A	G	-0.00111546	0.000198202	1.83×10^{-8}

17	3	rs6446264	49379810	A	C	-0.00111546	0.000198202	1.83×10^{-8}
18	3	rs35169793	49385841	G	A	-0.00111429	0.00019818	1.89×10^{-8}
19	3	rs6793308	49386850	T	C	-0.0011079	0.000198194	2.28×10^{-8}
20	3	rs111903592	49389886	C	G	-0.00111429	0.00019818	1.89×10^{-8}
21	3	rs773674547*	49391389	C	CA	-0.00115226	0.00020009	8.50×10^{-9}
22	3	rs11716974	49394420	A	G	-0.00111873	0.000198219	1.67×10^{-8}
23	3	rs71627385*	49395093	CA	C	-0.00110644	0.000198588	2.53×10^{-8}
24	3	rs9878943	49397221	A	G	-0.00111351	0.000198172	1.93×10^{-8}
25	3	rs543866446*	49398203	TAGCCAAGTG	T	-0.00110081	0.000198718	3.04×10^{-8}

26	3	rs9871380	49400788	A	G	-0.00111469	0.000198187	1.87×10^{-8}
27	3	rs11706370	49403658	A	G	-0.0011183	0.00019821	1.69×10^{-8}
28	3	rs60933862*	49408664	GTATA	G	-0.00112071	0.000198296	1.59×10^{-8}
29	3	rs6779524	49413016	T	C	-0.0011146	0.000198163	1.86×10^{-8}
30	3	rs6997	49416401	T	C	-0.00111953	0.000198178	1.62×10^{-8}
31	3	rs9814873	49416679	G	A	-0.00112336	0.00019822	1.46×10^{-8}
32	3	rs10640	49416844	A	G	-0.00111926	0.000198208	1.64×10^{-8}
33	3	rs11715915	49417897	T	C	-0.00112259	0.00019822	1.49×10^{-8}
34	3	rs9859556	49418553	T	G	-0.00112179	0.000198219	1.52×10^{-8}

35	3	rs554973188*	49418668	AT	A	-0.00111657	0.000198253	1.79×10^{-8}
36	3	rs11922013	49420922	C	G	-0.00111336	0.000198168	1.93×10^{-8}
37	3	rs6446272	49425854	A	G	-0.00111157	0.000198182	2.04×10^{-8}
38	3	rs7646366	49433235	A	G	-0.00111406	0.000198219	1.91×10^{-8}
39	3	rs6446277	49435644	A	G	-0.00110631	0.000198112	2.35×10^{-8}
40	3	rs35115732	49437722	C	T	-0.00111847	0.000198207	1.68×10^{-8}
41	3	rs13079643	49440033	A	G	-0.00111588	0.000198191	1.80×10^{-8}
42	3	3:49478988_CT_C*	49441555	C	CT	-0.00110657	0.000198497	2.49×10^{-8}
43	3	rs71324962	49450054	C	A	-0.0011165	0.000198168	1.77×10^{-8}

44	3	3:49489827_CA_C*	49452394	C	CA	-0.00115445	0.000200264	8.21×10^{-9}
45	3	rs34067452*	49452755	AT	A	-0.00109856	0.00019839	3.08×10^{-8}
46	3	rs9841110	49455048	G	C	-0.00111488	0.00019845	1.94×10^{-8}
47	3	rs67216675	49455718	T	G	-0.00111178	0.000198442	2.12×10^{-8}
48	3	rs6767355	49466662	C	T	-0.00112117	0.000198236	1.56×10^{-8}
49	3	rs9853683	49475155	G	A	-0.0011121	0.000198442	2.10×10^{-8}
50	3	rs12330269	49476477	A	G	-0.0011121	0.000198442	2.10×10^{-8}
51	3	rs9827021	49477331	A	C	-0.00110762	0.000198412	2.38×10^{-8}
52	3	rs34893377*	49478539	TA	T	-0.00114799	0.000200988	1.12×10^{-8}

53	3	rs7630869	49485110	T	C	-0.00110193	0.000198437	2.82×10^{-8}
54	3	rs9818590	49487663	G	A	-0.00110685	0.000198412	2.43×10^{-8}
55	3	rs11719996	49488525	C	T	-0.00110729	0.000198458	2.42×10^{-8}
56	3	rs9850072	49492049	T	C	-0.00110516	0.000198475	2.58×10^{-8}
57	3	rs138073019*	49498696	TTTA	T	-0.00110872	0.000199342	2.68×10^{-8}
58	3	rs34363169	49500279	A	G	-0.00110582	0.000198458	2.53×10^{-8}
59	3	rs35261698	49500406	G	C	-0.00110436	0.000198432	2.62×10^{-8}
60	3	rs6766131	49501499	C	T	-0.00110436	0.000198432	2.62×10^{-8}
61	3	rs6797664	49501789	A	G	-0.00110582	0.000198458	2.53×10^{-8}

62	3	rs6766581	49501968	C	T	-0.00110582	0.000198458	2.53×10^{-8}
63	3	rs11708955	49502681	C	T	-0.0011029	0.000198052	2.57×10^{-8}
64	3	rs11712569	49502719	G	A	-0.00110496	0.000198464	2.59×10^{-8}
65	3	rs10212296	49504840	C	T	-0.00110702	0.000198479	2.45×10^{-8}
66	3	rs9858418	49505855	G	A	-0.00110436	0.000198432	2.62×10^{-8}
67	3	rs9821311	49506223	T	C	-0.00110436	0.000198432	2.62×10^{-8}
68	3	rs7622302	49510128	C	T	-0.0011094	0.000198479	2.28×10^{-8}
69	3	rs7633271	49518530	C	T	-0.00111117	0.000198459	2.16×10^{-8}
70	3	rs3926569	49521283	T	C	-0.00111265	0.000198445	2.07×10^{-8}
71	3	rs13062429	49522052	A	G	-0.0011201	0.000198438	1.66×10^{-8}

72	3	rs34196454	49526023	A	G	-0.00111713	0.000198491	1.83×10^{-8}
73	3	rs1801143	49532767	T	C	-0.00111791	0.000198464	1.78×10^{-8}
74	3	rs4625	49534707	G	A	-0.00109923	0.000198177	2.92×10^{-8}
75	3	rs13079082	49537375	G	C	-0.00109186	0.00019773	3.36×10^{-8}
76	3	rs11130203	49537934	A	G	-0.0010952	0.000197637	3.01×10^{-8}
77	3	3:49577046_TAG_T*	49539613	T	TAG	-0.00109532	0.000197664	3.01×10^{-8}
78	3	rs34293138	49541584	C	T	-0.00109979	0.000197647	2.64×10^{-8}
79	3	rs3811697	49553337	T	C	-0.00109976	0.000197652	2.64×10^{-8}
80	3	rs9837027	49559580	T	C	-0.00109434	0.000197645	3.09×10^{-8}

81	3	rs35999162	49559797	G	C	-0.00110476	0.000197801	2.34×10^{-8}
82	3	rs9858280	49560304	C	T	-0.00110404	0.000197685	2.35×10^{-8}
83	3	rs11926781	49565856	T	C	-0.00111791	0.000197737	1.58×10^{-8}
84	3	rs34427167	49572790	T	C	-0.00111227	0.000197724	1.86×10^{-8}
85	3	rs11713474	49574024	G	A	-0.00111129	0.000197699	1.90×10^{-8}
86	3	3:49611660_CA_C*	49574227	C	CA	-0.00111092	0.000197716	1.93×10^{-8}
87	3	rs71324979	49582218	C	T	-0.0011168	0.000197738	1.63×10^{-8}
88	3	rs9811982	49586944	C	A	-0.00111358	0.000197701	1.78×10^{-8}
89	3	rs36106925*	49589844	GT	G	-0.00111358	0.000202158	3.63×10^{-8}

90	3	rs9875617	49597263	A	G	-0.00111395	0.000197729	1.77×10^{-8}
91	3	rs11720597	49597921	A	G	-0.00112959	0.00019888	1.35×10^{-8}
92	3	3:49638084_AAAATT_A*	49600651	A	AAAATT	-0.00113867	0.000202548	1.90×10^{-8}
93	3	rs13064576	49604997	T	C	-0.00111198	0.000199299	2.42×10^{-8}
94	3	rs11917431	49606579	T	C	-0.00111789	0.000199326	2.05×10^{-8}
95	3	rs11921590	49606760	C	T	-0.00111198	0.000199299	2.42×10^{-8}
96	3	rs9827708	49612556	G	C	-0.00111493	0.000199122	2.16×10^{-8}
97	3	rs7614725	49612962	A	G	-0.00111493	0.000199122	2.16×10^{-8}
98	3	rs9833611	49613777	A	G	-0.00111557	0.000199123	2.12×10^{-8}

99	3	3:49654710_AATACATTATATATATATATAT_A*	49617277	A	AATACATTATATATATATAT	-0.00114067	0.000199485	1.08×10^{-8}
100	3	rs9837341	49627334	G	A	-0.00111476	0.000199145	2.18×10^{-8}
101	3	rs6803222	49628543	G	T	-0.00111571	0.000199136	2.12×10^{-8}
102	3	rs1873625	49629531	A	C	-0.00111904	0.000199149	1.93×10^{-8}
103	3	rs9824092	49636714	G	C	-0.00111941	0.000199171	1.91×10^{-8}
104	3	rs9862080	49637025	G	A	-0.0011181	0.000199178	1.99×10^{-8}
105	3	rs11710037	49637901	G	C	-0.00112019	0.000199185	1.87×10^{-8}
106	3	rs2172252	49640874	T	A	-0.001115	0.000200718	2.78×10^{-8}
107	3	rs4283605	49641218	G	A	-0.001115	0.000200682	2.77×10^{-8}

108	3	rs9812791	49644878	A	G	-0.00110799	0.000200711	3.39×10^{-8}
109	3	rs13316695	49647484	G	C	-0.00111283	0.000200715	2.96×10^{-8}
110	3	rs6770670	49649249	C	T	-0.00114274	0.000200941	1.30×10^{-8}
111	3	rs34762726	49651777	A	G	-0.00114264	0.000200931	1.30×10^{-8}
112	3	rs11709525	49653063	T	C	-0.0011428	0.000200986	1.30×10^{-8}
113	3	rs11718165	49659364	G	A	-0.00111982	0.000200726	2.43×10^{-8}
114	3	rs9836291	49660026	A	G	-0.00111142	0.000200659	3.05×10^{-8}
115	3	rs9858542	49664550	A	G	-0.00112092	0.000200692	2.34×10^{-8}
116	3	rs9882740	49665051	C	T	-0.00112897	0.0002008	1.89×10^{-8}

117	3	rs9823546	49668079	A	T	-0.00112621	0.000200776	2.04×10^{-8}
118	3	rs13093385	49673046	T	A	-0.00111819	0.000200717	2.54×10^{-8}
119	3	3:49710514_CCGGG_C*	49673081	C	CCGGG	-0.00125187	0.000207697	1.67×10^{-9}
120	3	rs11130213	49674864	T	C	-0.00111888	0.000200727	2.49×10^{-8}
121	3	rs1131095	49676792	C	T	-0.00111847	0.000200726	2.52×10^{-8}
122	3	rs9822268	49682296	A	G	-0.00110701	0.00020074	3.50×10^{-8}
123	3	rs3197999	49684099	A	G	-0.00113243	0.000202288	2.17×10^{-8}
124	3	rs13085791	49684365	A	C	-0.00111823	0.000200779	2.56×10^{-8}
125	3	rs9852529	49693192	A	G	-0.00111719	0.000200866	2.68×10^{-8}

126	3	rs9858213	49694428	T	G	-0.00111584	0.00020084	2.77×10^{-8}

CARE = circadian activity rhythm energy, CHR = chromosome, GWAS = genome-wide association study, SNP = single nucleotide polymorphism, position = base pair coordinate hg38, A1/A2 = effect/non-effect alleles, SE = standard error. This table presents summary statistics for 126 variants associated with CARE (significance level for genome-wide association test was set at $P < 5\times10^{-8}$) in the adult dataset of UK Biobank. Out of these, 109 instrumental variables were used in the Mendelian randomization analysis, while 17 variants removed were annotated with an asterisk.

Supplementary Table 10. Significant variants associated with relative amplitude ($P < 5 \times 10^{-8}$) in the adult dataset (UK Biobank).

SNP	Chr:position	Nearest gene(s)	Alleles (E/A)	BETA	SE	*P* value
**rs1110666**	22:17974954	MICAL3	G/T	0.002	0.0004	2.01×10^{-8}
rs9605481	22: 17982911	MICAL3, MIR648	A/G	0.002	0.0004	2.29×10^{-8}
rs12157484	22: 17972356	MICAL3	T/C	0.002	0.0004	2.67×10^{-8}

Chr = chromosome, E/A = effect/non-effect alleles, position = base pair coordinate hg38, SE = standard error, SNP = single nucleotide polymorphism. Genetic association analysis was performed in related subjects of European ancestry using linear regression models adjusted for age, sex, 20 principal components of ancestry, genotyping array, and genetic correlation matrix. Lead variants in each locus are shown in bold. Genes indicate all genes within the locus of interest.

Supplementary Table 11. Mendelian randomization analysis for CARE and cognitive functions using GWAS summary statistics in the adult dataset (UK Biobank).

Exposure	Outcome	Method	n SNPs	Beta	SE	*P* value
CARE	Reasoning ability	Weighted median	109	-59.91	1.53	<0.0001
		MR-Lasso	109	-59.6	0.76	<0.0001
		MBE	109	-60.15	3.96	<0.0001
		MR-cML	109	-59.4	1.22	<0.0001
		Inverse variance weighted	109	-59.6	0.76	<0.0001
CARE	Short-term memory	Weighted median	109	7.94	0.53	<0.0001
		MR-Lasso	109	7.51	0.4	<0.0001
		MBE	109	8.03	1.4	<0.0001
		MR-cML	109	7.5	0.45	<0.0001
		Inverse variance weighted	109	7.51	0.4	<0.0001
CARE	Prospective memory	Weighted median	109	16.85	1.23	<0.0001
		MR-Lasso	109	16.97	0.94	<0.0001
		MBE	109	16.67	3.12	<0.0001
		MR-cML	109	16.93	1.04	<0.0001
		Inverse variance weighted	109	16.97	0.94	<0.0001

CARE = circadian activity rhythm energy, GWAS = genome-wide association study, MR = mendelian randomization, SE = standard error. Mendelian randomization analyses were performed to examine the causal relationship between CARE and cognitive functions, and the significant level was set at $P < 0.017$.

Supplementary Table 12. Significant gene-CARE associations from single-tissue enrichment analysis using UTMOST in 44 GTEx tissues.

Index	Tissue	Gene	z-score	*P* value
1	Adipose_Subcutaneous	APEH	-5.663808082	1.48×10^{-8}
2	Adipose_Subcutaneous	SEMA3F	-5.302601219	1.14×10^{-7}
3	Adipose_Subcutaneous	TMEM89	-4.956267282	7.19×10^{-7}
4	Adipose_Subcutaneous	MST1R	4.944310489	7.64×10^{-7}
5	Adipose_Subcutaneous	QARS	4.692930945	2.69×10^{-6}
6	Adipose_Visceral_Omentum	APEH	-5.651974844	1.59×10^{-8}
7	Adipose_Visceral_Omentum	DOCK3	4.87406258	1.09×10^{-6}
8	Adipose_Visceral_Omentum	SEMA3F	-4.762938165	1.91×10^{-6}
9	Adrenal_Gland	SEMA3F	-5.625818817	1.85×10^{-8}
10	Artery_Aorta	TRAIP	6.194041916	5.86×10^{-10}
11	Artery_Aorta	APEH	-5.704117795	1.17×10^{-8}
12	Artery_Aorta	MST1	-5.656919047	1.54×10^{-8}
13	Artery_Aorta	PRPF3	5.513689629	3.51×10^{-8}
14	Artery_Aorta	MST1R	4.980493922	6.34×10^{-7}
15	Artery_Aorta	CDHR4	4.966337126	6.82×10^{-7}
16	Artery_Aorta	HYAL1	4.832886368	1.35×10^{-6}
17	Artery_Coronary	APEH	-5.583866777	2.35×10^{-8}
18	Artery_Coronary	MAPKAPK3	-5.296424557	1.18×10^{-7}
19	Artery_Coronary	CELSR3	5.230057	1.69×10^{-7}
20	Artery_Tibial	GPX1	5.667087334	1.45×10^{-8}
21	Artery_Tibial	APEH	-5.658779713	1.52×10^{-8}
22	Artery_Tibial	TMEM89	-5.335339202	9.54×10^{-8}
23	Artery_Tibial	MST1R	4.905785815	9.31×10^{-7}
24	Brain_Anterior_cingulate_cortex_BA24	APEH	-5.27939848	1.30×10^{-7}
25	Brain_Anterior_cingulate_cortex_BA24	IP6K1	-4.827449664	1.38×10^{-6}

26	Brain_Anterior_cingulate_cortex_BA24	GPX1	4.817048873	1.46×10^{-6}
27	Brain_Caudate_basal_ganglia	USP4	-5.545881985	2.92×10^{-8}
28	Brain_Caudate_basal_ganglia	MST1	-5.28132285	1.28×10^{-7}
29	Brain_Caudate_basal_ganglia	MST1R	5.003170012	5.64×10^{-7}
30	Brain_Cerebellar_Hemisphere	GPX1	5.366869786	8.01×10^{-8}
31	Brain_Cerebellar_Hemisphere	DOCK3	-5.021291687	5.13×10^{-7}
32	Brain_Cerebellar_Hemisphere	ZFP69B	-4.770887501	1.83×10^{-6}
33	Brain_Cerebellum	CCDC36	4.77807275	1.77×10^{-6}
34	Brain_Cerebellum	TRAIP	4.774628689	1.80×10^{-6}
35	Brain_Cortex	QRICH1	-5.265106377	1.40×10^{-7}
36	Brain_Cortex	MST1R	5.241327041	1.59×10^{-7}
37	Brain_Cortex	FAM212A	-4.992799428	5.95×10^{-7}
38	Brain_Frontal_Cortex_BA9	TRAIP	4.942486012	7.71×10^{-7}
39	Brain_Frontal_Cortex_BA9	AMIGO3	4.661742966	3.14×10^{-6}
40	Brain_Hippocampus	APEH	-5.532191938	3.16×10^{-8}
41	Brain_Hippocampus	RNF123	4.859485898	1.18×10^{-6}
42	Brain_Hypothalamus	MST1	-5.629418272	1.81×10^{-8}
43	Brain_Hypothalamus	USP4	-5.344681433	9.06×10^{-8}
44	Brain_Hypothalamus	TRAIP	5.177950263	2.24×10^{-7}
45	Brain_Hypothalamus	FAM212A	-5.002924245	5.65×10^{-7}
46	Brain_Hypothalamus	QARS	-4.977952658	6.43×10^{-7}
47	Brain_Hypothalamus	DOCK3	4.962955014	6.94×10^{-7}
48	Brain_Hypothalamus	SEMA3F	-4.728706923	2.26×10^{-6}
49	Brain_Nucleus_accumbens_basal_ganglia	DOCK3	-4.81294507	1.49×10^{-6}
50	Brain_Nucleus_accumbens_basal_ganglia	MST1R	4.700843784	2.59×10^{-6}
51	Brain_Putamen_basal_ganglia	DOCK3	5.260604407	1.44×10^{-7}
52	Breast_Mammary_Tissue	APEH	-5.565385208	2.62×10^{-8}
53	Cells_EBV-transformed_lymphocytes	APEH	-5.639306777	1.71×10^{-8}
54	Cells_EBV-transformed_lymphocytes	TRAIP	-5.049518852	4.43×10^{-7}
55	Cells_EBV-transformed_lymphocytes	USP4	5.046117578	4.51×10^{-7}

56	Cells_Transformed_fibroblasts	TRAIP	-6.219676938	4.98×10^{-10}
57	Cells_Transformed_fibroblasts	APEH	-5.666230315	1.46×10^{-8}
58	Cells_Transformed_fibroblasts	MAPKAPK3	-5.359752371	8.33×10^{-8}
59	Colon_Sigmoid	DOCK3	-5.422599827	5.87×10^{-8}
60	Colon_Sigmoid	SEMA3F	-4.730694545	2.24×10^{-6}
61	Colon_Transverse	C3orf18	5.280804566	1.29×10^{-7}
62	Esophagus_Gastroesophageal_Junction	SEMA3F	-5.651459817	1.59×10^{-8}
63	Esophagus_Gastroesophageal_Junction	TRAIP	5.277078021	1.31×10^{-7}
64	Esophagus_Gastroesophageal_Junction	MAPKAPK3	4.961722819	6.99×10^{-7}
65	Esophagus_Gastroesophageal_Junction	MST1R	4.77542302	1.79×10^{-6}
66	Esophagus_Gastroesophageal_Junction	SLC26A6	-4.675947245	2.93×10^{-6}
67	Esophagus_Mucosa	MST1	-6.329424121	2.46×10^{-10}
68	Esophagus_Mucosa	APEH	-5.618707	1.92×10^{-8}
69	Esophagus_Mucosa	MAPKAPK3	-5.600475301	2.14×10^{-8}
70	Esophagus_Mucosa	KLHDC8B	5.052016996	4.37×10^{-7}
71	Esophagus_Muscularis	APEH	-5.31284547	1.08×10^{-7}
72	Heart_Atrial_Appendage	C3orf18	5.757714446	8.53×10^{-9}
73	Heart_Atrial_Appendage	TRAIP	5.294556686	1.19×10^{-7}
74	Heart_Atrial_Appendage	MAPKAPK3	-5.133247136	2.85×10^{-7}
75	Heart_Left_Ventricle	C3orf18	4.749331667	2.04×10^{-6}
76	Liver	MST1R	-5.185892834	2.15×10^{-7}
77	Lung	TRAIP	5.026221416	5.00×10^{-7}
78	Lung	MST1	-4.936358522	7.96×10^{-7}
79	Lung	USP4	4.80733741	1.53×10^{-6}
80	Lung	UBA7	-4.783035373	1.73×10^{-6}
81	Muscle_Skeletal	USP4	5.259665486	1.44×10^{-7}
82	Nerve_Tibial	APEH	-5.590508735	2.26×10^{-8}

83	Nerve_Tibial	SEMA3F	-5.000711045	5.71×10^{-7}
84	Ovary	FAM212A	5.492151061	3.97×10^{-8}
85	Ovary	MAPKAPK3	5.161171174	2.45×10^{-7}
86	Ovary	IMPDH2	-5.14428585	2.69×10^{-7}
87	Ovary	APEH	-5.041715118	4.61×10^{-7}
88	Ovary	TRAIP	4.725188338	2.30×10^{-6}
89	Pancreas	IMPDH2	-5.427060124	5.73×10^{-8}
90	Pancreas	APEH	-5.343133577	9.14×10^{-8}
91	Pancreas	HYAL1	-4.873398869	1.10×10^{-6}
92	Pituitary	MAPKAPK3	-5.152055795	2.58×10^{-7}
93	Prostate	C3orf18	5.368373385	7.94×10^{-8}
94	Prostate	C3orf62	5.180126404	2.22×10^{-7}
95	Prostate	USP4	4.924631701	8.45×10^{-7}
96	Skin_Not_Sun_Exposed_Suprapubic	MST1	-5.973310792	2.32×10^{-9}
97	Skin_Not_Sun_Exposed_Suprapubic	APEH	-5.644251054	1.66×10^{-8}
98	Skin_Not_Sun_Exposed_Suprapubic	USP4	5.622727937	1.88×10^{-8}
99	Skin_Not_Sun_Exposed_Suprapubic	RNF123	4.773239505	1.81×10^{-6}
100	Skin_Not_Sun_Exposed_Suprapubic	TRAIP	4.769536346	1.85×10^{-6}
101	Skin_Sun_Exposed_Lower_leg	APEH	-5.606026796	2.07×10^{-8}
102	Skin_Sun_Exposed_Lower_leg	TUSC2	-5.136524997	2.80×10^{-7}
103	Skin_Sun_Exposed_Lower_leg	MST1	-4.990475528	6.02×10^{-7}
104	Small_Intestine_Terminal_Ileum	C3orf18	5.08643784	3.65×10^{-7}
105	Spleen	MST1	-5.583443803	2.36×10^{-8}
106	Spleen	GMPPB	5.384605492	7.26×10^{-8}
107	Spleen	IMPDH2	5.253539725	1.49×10^{-7}
108	Spleen	AMIGO3	5.154687721	2.54×10^{-7}
109	Spleen	NICN1	-5.124124587	2.99×10^{-7}
110	Spleen	FAM212A	-4.988635993	6.08×10^{-7}

111	Spleen	SEMA3F	-4.874096876	1.09×10^{-6}
112	Spleen	GPX1	4.765705173	1.88×10^{-6}
113	Stomach	COL7A1	5.563891411	2.64×10^{-8}
114	Testis	MST1R	4.940485038	7.79×10^{-7}
115	Thyroid	FAM212A	-5.529029428	3.22×10^{-8}
116	Thyroid	USP4	5.318316636	1.05×10^{-7}
117	Thyroid	MAPKAPK3	4.900567226	9.56×10^{-7}
118	Whole_Blood	APEH	-5.554013462	2.79×10^{-8}
119	Whole_Blood	MAPKAPK3	-5.361156159	8.27×10^{-8}

CARE = circadian activity rhythm energy. The Bonferroni-corrected threshold for significance was 2.9×10^{-6} for single-tissue gene-CARE association tests using UTMOST.

Supplementary Table 13. Significant genes associated with CARE detected by cross-tissue transcriptome-wide association analysis using UTMOST in 44 GTEx tissues.

Gene	Test score	*P* value
QARS	14.29695	3.2×10^{-7}
SEMA3F	14.19545	3.10×10^{-7}
TRAIP	17.88998	7.28×10^{-9}
USP4	13.72578	6.88×10^{-7}
COL7A1	13.0528	1.42×10^{-6}
DOCK3	12.91689	1.40×10^{-6}
FAM212A	13.2909	2.24×10^{-6}
GPX1	12.51486	8.85×10^{-6}
APEH	17.2256	9.14×10^{-9}
C3orf18	12.95729	9.04×10^{-7}
IMPDH2	16.09161	7.03×10^{-8}
MAPKAPK3	17.61598	1.67×10^{-8}
MST1	16.40505	1.83×10^{-8}

CARE = circadian activity rhythm energy, GTEx = the Genotype-Tissue Expression project, UTMOST = Unified Test for MOlecular SignaTures. The Bonferroni-corrected threshold for significance was 2.9×10^{-6} for cross-tissue gene-CARE association tests using UTMOST.
